# Doublecortin in the Fish Visual System, a Specific Protein of Maturing Neurons

**DOI:** 10.3390/biology11020248

**Published:** 2022-02-06

**Authors:** Laura DeOliveira-Mello, Isabel Vicente, Veronica Gonzalez-Nunez, Adrian Santos-Ledo, Almudena Velasco, Rosario Arévalo, Juan M. Lara, Andreas F. Mack

**Affiliations:** 1 Institute of Neurosciences of Castilla and León, University of Salamanca, 37007 Salamanca, Spain; vgnunez@usal.es (V.G.-N.); santosledo@usal.es (A.S.-L.); malmu@usal.es (A.V.); mraa@usal.es (R.A.); rororo@usal.es (J.M.L.); 2 Institute for Biomedical Research of Salamanca (IBSAL), University of Salamanca, 37007 Salamanca, Spain; 3 Department of Agriculture, Food and Environment, University of Pisa, 56124 Pisa, Italy; i.vicentemu@gmail.com; 4 Institute of Clinical Anatomy and Cell Analysis, Eberhard-Karls Universität Tübingen, 72074 Tübingen, Germany; an.mack@uni-tuebingen.de

**Keywords:** doublecortin, visual system, *Danio rerio*, *Astatotilapia burtoni*, neurogenesis

## Abstract

**Simple Summary:**

Doublecortin (DCX) is an essential protein in the development of the central nervous system and in lamination of the mammalian cortex. It is known that the expression of DCX is restricted to newborn neurons. The visual system of teleost fish has been postulated as an ideal model since it continuously grows throughout the animal’s life. Here, we report a comparative expression analysis of DCX between two teleost fish species as well as a bioinformatic analysis with other animal groups. Our results demonstrate that DCX is very useful for identifying new neurons in the visual systems of *Astatotilapia burtoni*, but is absent in *Danio rerio*.

**Abstract:**

Doublecortin (DCX) is a microtubule associated protein, essential for correct central nervous system development and lamination in the mammalian cortex. It has been demonstrated to be expressed in developing—but not in mature—neurons. The teleost visual system is an ideal model to study mechanisms of adult neurogenesis due to its continuous life-long growth. Here, we report immunohistochemical, *in silico*, and western blot analysis to detect the DCX protein in the visual system of teleost fish. We clearly determined the expression of DCX in newly generated cells in the retina of the cichlid fish *Astatotilapia burtoni*, but not in the cyprinid fish *Danio rerio*. Here, we show that DCX is not associated with migrating cells but could be related to axonal growth. This work brings to light the high conservation of DCX sequences between different evolutionary groups, which make it an ideal marker for maturing neurons in various species. The results from different techniques corroborate the absence of DCX expression in zebrafish. In *A. burtoni*, DCX is very useful for identifying new neurons in the transition zone of the retina. In addition, this marker can be applied to follow axons from maturing neurons through the neural fiber layer, optic nerve head, and optic nerve.

## 1. Introduction

Doublecortin (DCX; also known as doublin or lissencephalin-X) is a microtubule-associated protein which is typically expressed in the early neuronal differentiation stage, both in precursors and immature neurons [1,2]. Due to DCX expression being nearly exclusive to developing neurons, several research groups are using it as a marker for neurogenesis in a wide range of vertebrate species, e.g., mammals [3], lampreys [4], sharks [5], and teleosts [6].

Brain formation depends on microtubules (MTs) and accompanying microtubule associated proteins (MAPs) to regulate specific migration of different neural cell types [7]. Brain development includes nuclear displacement and process formation that require the action of MTs and specific MAPs [8]. Furthermore, MTs are essential in the formation of growth cones [9]. The de-stabilization of MTs leads to the collapse of the migrating cell body and cessation of nuclear translocation [10]. Faulty of DCX expression causes critical brain defects, which implies that other MTs stabilizing proteins cannot compensate for DCX function in the central nervous system (CNS) [7,11]. In newly formed neurons, DCX are involved in the growth of neuronal processes [12,13].

The continuous life-long growth of the visual system of anamniotes (such as teleost fish) has been an intriguing phenomenon [14,15], especially since such extensive growth does not occur in mammals [16,17]. Several studies have used different fish species to understand the mechanism of adult neurogenesis in vertebrates [2,18,19]. The retina has been proposed as an ideal model to study the generation of new neurons in adults due to the presence of well-delimited neurogenic zones [20,21]: the peripheral germinal zone (PGZ), formed by stem cells; the transition zone, occupied by differentiating cells; and the layered retina, harboring completely differentiated cells, except for the generation of new rods which are added to the outer nuclear layer from rod precursors [22]. Among the many markers used to label differentiating neurons, DCX stands out both in mammals and some teleosts such as cichlid fish [6,23]. Given that commercially available anti-DCX-antibodies efficiently label processes from maturing neurons [12], we were interested in testing DCX as a potential marker to study differentiating neurons within the retinal transition zone of the fish retina.

The aim of the present study was to identify newly generated DCX-positive neurons in the adult fish retina and to follow their axonal pathway into the optic nerve. We used two fish, the zebrafish (*Danio rerio*) and Burton’s mouthbrooder fish (*Astatotilapia burtoni*, formerly known as *Haplochromis burtoni*). The zebrafish is the main non-mammalian vertebrate animal model used in scientific research including visual studies [24], and *A. burtoni* is a model organism for behavior showing a substantial growth of retinal tissue throughout its life [15,25].

Here, we report the immunohistological reactivity for DCX in *A. burtoni* retina, which was conspicuously absent in the zebrafish retina. In addition, we performed *in silico* and western blotting analyses in order to clearly define the presence of DCX in different models—from flies to mice—to study neurogenesis.

## 2. Materials and Methods

### 2.1. Animals

We used adult specimens of *Astatotilapia burtoni* (Ostariophysi; *Cichlidae*) measuring between 2–6 cm standard length and *Danio rerio* (Acanthopterygii; *Cyprinidae*) measuring more than 1.5 cm. All animals were bred in our own animal facilities, in the Institute of Clinical Anatomy and Cell Analysis (University of Tübingen, Tübingen, Germany) and in the Institute of Neuroscience of Castilla y León (University of Salamanca, Salamanca, Spain). For protein expression assays, mice tissue was obtained from brains (excluding olfactory bulb and cerebellum) of newborn mice (C57BL/6 strain at p10) kindly provided by Dr. J.R. Alonso’s group of University of Salamanca [26].

Both fish species were kept at 28.5 ± 1 °C, with a photoperiod on 12 h light/dark cycle, and without feeding restrictions. All procedures were performed in accordance with the guidelines of the European Union Council Directive (2010/63/EU). Local authorities (Regierungspräsidium Tübingen and the Animal Ethical Committee of The University of Salamanca) approved animal use before experimentation in both institutions.

### 2.2. Tissue Preparation

We used the eyes and optic nerves from at least four animals of each fish species for histological analysis. Adult fish were deeply anaesthetized with tricaine methane sulphonate (MS222; Sigma-Aldrich, St. Louis, MO, USA, E10521) and sacrificed by decapitation. Samples were fixed in 4% paraformaldehyde dissolved in phosphate buffer saline 0.1M pH 7.2 (PBS) during overnight at 4 °C. After fixation, samples were rinsed in PBS and cryoprotected in 30% sucrose, overnighted at 4 °C, embedded in Tissue-Tek O.C.T. compound (Sakura, Tokyo, Japan, 4583) and sectioned on a cryostat at 14 μm thickness.

For tissue clearing, the entire eyeballs with a short piece of optic nerve were dissected for each species, removing the sclera and outer pigmented tissue. Before tissue clearing, we applied a bleaching protocol to eliminate pigment granules. Samples were incubated in a bleaching solution constituted of 0.5% (*v*/*v*) KOH, and 1% (*v*/*v*) Na_2_HPO_4_ in H_2_O plus 3% H_2_O_2_ (Sigma-Aldrich, St. Louis, MO, USA, H1009). Sequential incubations of two hours at room temperature were performed until sample bleaching was achieved, followed by washing in acid acetic for 1 h at room temperature. Then, we applied our adjusted clearing protocol [27]. Briefly, samples were fixed overnight in a hydrogel monomer solution constituted by 10% (*v*/*v*) acrylamide, 2.5% (*v*/*v*) bis-acrylamide, 4% (*wt/v*) paraformaldehyde, and 0.25% (*wt/v*) VA-044 Initiator (FUJIFILM Wako Pure Chemical, Neuss, Germany,, 017-19362) in PBS (pH 7.4). The gel was polymerized in a vacuum oven at 40–45 °C for 3 h. Embedded eyes were cleared in an 8% (*wt/v*) sodium dodecyl sulfate solution (SDS) in PBS at 45–50 °C for 1–2 weeks. Clearing solution was changed every 2 days.

### 2.3. Immunostaining

The cryosections were pre-incubated for 90 min at room temperature in PBS with 0.02% (*v*/*v*) Triton X100 (Sigma-Aldrich, St. Louis, MO, USA, X100-100ML), 5% (*v*/*v*) normal donkey serum (Jackson ImmunoResearch, West Grove, PA, USA) and 1% (*v*/*v*) dimethyl sulfoxide (DMSO; Sigma-Aldrich, St. Louis, MO, USA). For PCNA labeling, sections were treated with 2 N HCl in PBS for 30 min before pre-incubation. After blocking, samples were incubated with anti-DCX (1:100. Santa Cruz Biotechnology, Dallas, TX, USA; sc-8066) and anti-PCNA (1:300. Santa Cruz Biotechnology, Dallas, TX, USA; sc-56) antibodies diluted in PBS with 0.02% (*v*/*v*) Triton X, and 5% (*v*/*v*) normal donkey serum, and incubated overnight at 4 °C. After 3 washes for 10 min with PBS, sections were incubated in anti-goat Alexa 488 (1:400) conjugated antibody (Jackson ImmunoResearch, West Grove, PA, USA; 705-545-003) and anti-mouse Cy3 (1:250) conjugated antibody (Jackson ImmunoResearch, West Grove, PA, USA; 715-165-150) for 90 min at room temperature in PBS with 0.02% (*v*/*v*) Triton X, and 5% (*v*/*v*) normal donkey serum. Nuclei were labeled with DRAQ5 (Thermo Fisher Scientific, Waltham, MA, USA). Negative controls without first or secondary antibodies were included, and no staining was detected. Sections were mounted with Mowiol^®^ or Fluoromont^®^.

In the clearing samples, after washing SDS solution, we performed immunostains to detect DCX in entire eyes of teleost fish. Pre-incubation was performed overnight by using donkey serum. The following day, samples were incubated in anti-DCX primary antibody for 4 days followed by an overnight incubation with secondary antibody and DRAQ5. Several washing steps were performed to eliminate background fluorescence, followed by an incubation in 80% glycerol for refractive index matching.

### 2.4. Image Acquisition and Analysis

Images were acquired on an LSM Exciter confocal microscope (Zeiss, Jena, Germany) or an LSM510 using laser excitations at 488, 543, and 633 nm in sequential scans with appropriate sets. Images, as well as the three-dimensional reconstructions, were generated with ZEN 2009 software. Once adjusted for contrast and brightness, the image plates were assembled with Photoshop CS6.

### 2.5. Western Blotting

Total proteins were extracted from the brains of wild type adult zebrafish (AB strain), from adult *A. burtoni*, and from newborn mice (C57BL/6 strain at P10). Tissues were mechanically homogenized with hand-held sterile pestles and insulin syringes and lysed in RIPA buffer (50 mM Tris (VWR International Eurolab S.L, Barcelona, Spain, 0027C481) pH 8; 150 mM NaCl; 1% Igepal; 0.5% sodium deoxycholate; 0.1% SDS) containing a protease inhibitor cocktail (VWR; M222-1ML). Samples were centrifuged (10.000× *g* for 10 min at 4 °C) to eliminate the debris, and supernatants were transferred to clean Eppendorf tubes. Protein concentration was determined using the Bradford methodology.

Lysates were resuspended in 2× Laemmli buffer (4% SDS; 20% glycerol; 10% β-mercaptoethanol; 0.004% bromophenol blue; 0.125M TrisHCl pH 6.8) and boiled for 7 min. Amounts of 40 µg, 60 µg and 80 µg of total proteins were separated using conventional SDS-PAGE in reduced conditions and transferred to polyvinylidene difluoride membrane (PVDF, Amersham). Membranes were blocked with 3% bovine serum albumin (BSA) in TBS-T (Tris buffer; 0.1% Tween20, Sigma-Aldrich, St. Louis, MO, USA) for 1 h at room temperature and incubated with anti-DCX (Santa Cruz Biotechnology, Dallas, TX, USA; sc-8066) and anti-doublecortin-like kinase (DCLK; Abcam, Cambridge, UK, ab106639) 1:1.000 diluted in blocking buffer overnight at 4 °C. After 3 washes of 10 min each with TBS-T, membranes were incubated for 1 h at room temperature with the secondary antibody goat anti-rabbit conjugated with horseradish peroxidase (HRP) (1:10.000 dilution; Jackson InmunoResearch, West Grove, PA, USA; 111-035-003 Lot nº. 131599). Membranes were washed again, and HRP signal was developed using the chemoluminescence detection system (ECL; Advansta, San Jose, CA, USA, WesternBright™, 16101705) in MicroChemi 4.2 (DNR Bio-Imaging Systems). For the stripping procedure, membranes were washed in sequential incubations for 10 min each at room temperature in TBS-T, Gly 0.1 M (pH = 2), 1% SDS, and TBS-T. β-actin antibody (1:1.000 diluted in blocking buffer; Cell Signalling, Danvers, USA, 4697. Lot nº. 10/2016) was used as loading control.

Protein expression was determined by densitometric analysis using Adobe Photoshop CS6. Total integrated density was obtained for each band (DCX, DCLK, and β-actin) and background integrated density was subtracted to obtain the specific integrated density for each protein.

### 2.6. In Silico Identification and Analysis of DCX in Teleost Fish

Amino acid sequences of DCX and DCLK proteins of teleost fish were obtained by BLASTp analysis using the *Homo sapiens* DCX (Accession number: O43602) and DCLK (Accession number: AAI52457) protein sequences as queries against the non-redundant protein database of the National Center of Biotechnology Information (NCBI; Available online: www.ncbi.nlm.nih.gov (accessed on 19 April 2021).

In order to set up an accurate protein database, a two-parameter approach was followed: (i) exclusion of all DCX conserved domain-containing protein (DCDC) sequences, and (ii) selection of those proteins containing both the DCX1 (IPR003533; PF03607; cd16109) and DCX2 (IPR003533; PF03607; cd17069) conserved domains identified using InterPro Scan v5.44-79.0 [28] and the NCBI Conserved Domain Database. Finally, a total of 159 proteins were included in the database, containing all the sequences described by Reiner et al. [29] and those obtained here by BLASTp analysis.

Visualization and extraction of DCX1 or DCX2 conserved domains (Figure S1) were performed using Jalview v2.11.1.4 [30]. Multiple-sequence alignments of either the entire proteins or only DCX1 and DCX2 conserved domains were performed separately using MAFFT v7.450 [31].

We performed a tblastn analysis to detect *dcx* and *dclk* sequences in the genomes of two teleost fish: *Danio rerio* [Genome assembly number: GCA_000002035.4_GRCz11; [32]] and *Astatotilapia burtoni* [Genome assembly number: GCF_000239415.1_AstBur1.0; [33]], available at the NCBI database. The genomes of *Drosophila melanogaster* (Genome assembly number: GCA_000001215.4; [34]), and *Caenorhabditis elegans* (Genome assembly number: GCA_000002985.3; [35]) were used as controls. The DCX protein sequences and DCLK isoforms (DCLK1_ID: ENSG00000133083; DCLK2_ID: ENSG00000170390; DCLK3_ID: ENSG00000163673) from human available at the ENSEMBL database (Available online: www.ensembl.org (accessed on 25 July 2021)) were used as queries to search for *dcx* and *dclk* orthologous genes in the genomes of *D. rerio*, *A. burtoni*, *D. melanogaster* and *C. elegans*.

## 3. Results

### 3.1. DCX Is Present in A. burtoni Retina but Not D. rerio

**Figure 1:**
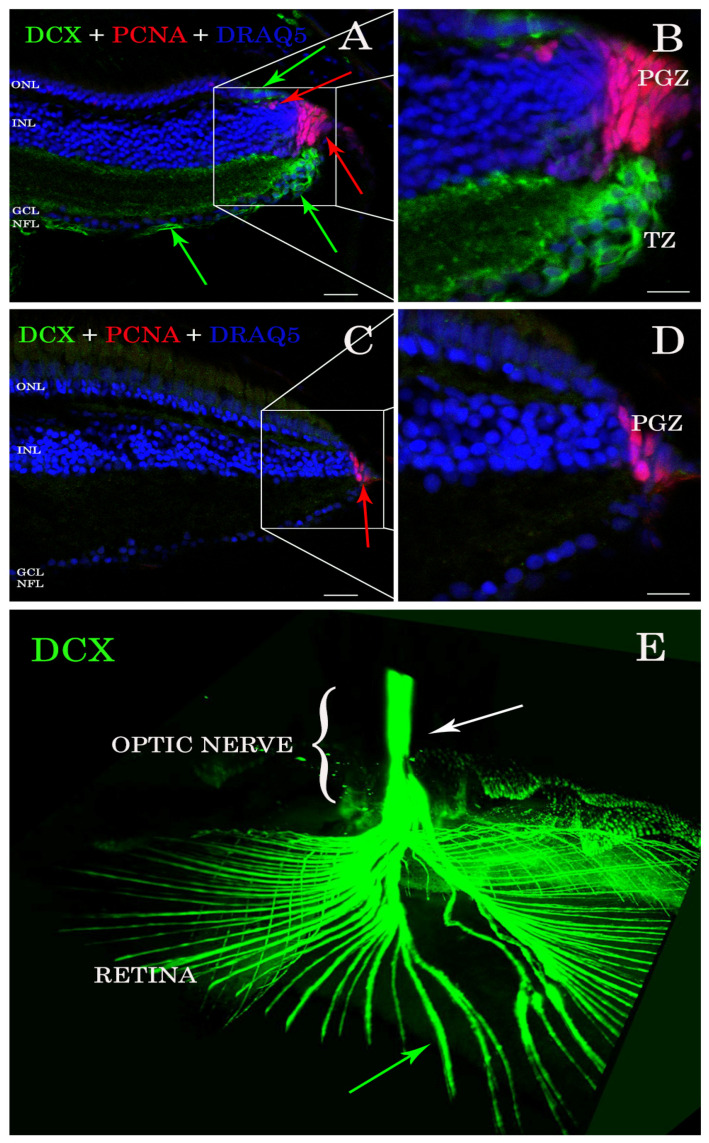
Sections of retina (**A**–**D**) and cleared tissue (**E**). DCX positive cells (green arrows) are detected in *A. burtoni* retina (**A**) but not in zebrafish (**C**). Magnification of (**A**,**C**) are presented in (**B**,**D**). The proliferating cell nuclear antigen (PCNA; red arrows) binding antibodies detect cells in the peripheral germinal zone (PGZ; of both species. In addition, DCX (green arrows) and PCNA (red arrows) are detected in the ONL corresponding to cones and rod precursors, respectively. Nuclei are stained with DRAQ5 (blue). No double positive cells for DCX (green arrows) and PCNA (red arrows) are found. By analyzing the whole transparent eyeball, it is possible to follow DCX positive processes in the neural fiber layer (green arrow) into the optic nerve (**E**; white arrow). Scale bar 20 µm (**A**,**C**); 10 µm (**B**,**D**). ONL: outer nuclear layer; INL: inner nuclear layer; GCL: ganglion cell layer; NFL: neural fiber layer.

Antibody stains applied to histological sections enabled the detection of DCX expression in the *A. burtoni* retina (Figure 1A,B). In contrast, no specific immunofluorescence was found in the zebrafish retina (Figure 1C,D). DCX positive cell bodies were located in the transition zone (Figure 1A,B), next to the PGZ, of the retina of *A. burtoni* and their axons passing through the nerve fiber layer to the optic nerve head (Figure 1E). No cell bodies expressing DCX were observed in the differentiated retina and/or in the optic nerve.

### 3.2. DCX Proteins Are Present in All the Genomes Analyzed but Not in Zebrafish

We generated a database containing 159 protein sequences belonging to 14 teleost fish (*Triplophysa tibetana*, *Danio rerio*, *Betta splendens*, *Parambassis ranga*, *Oryzias latipes*, *Nothobranchius furzeri*, *Astatotilapia burtoni*, *Gadus morhua*, *Anabarilius grahami*, *Nothobranchius pienaari*, *Oryzias melastigma*, *Carassius auratus*, *Labeo rohita*, and *Tetraodon nigroviridis*), 6 mammals (*Mus musculus*, *Homos sapiens*, *Pan troglodytes*, *Canis lupus*, *Bos taurus*, and *Rattus norvegicus*), one bird (*Gallus gallus*), and one shark (*Callorhinchus milii*), as well as *D*. *melanogaster* and *C*. *elegans* as invertebrate species (Table 1).

Alignment of DCX amino acidic sequences showed a high conservation degree within and among analyzed groups (Table 2). Interestingly, shark and human DCX homologues share 96% of similarity, but no DCX coding sequence was found in the genome of zebrafish.

Since we could not find DCX in zebrafish, we also considered the DCLK proteins, which have been previously described in zebrafish, and belong to the DCX superfamily [29]. DCLK sequences showed around 50–55% pairwise identity among species (Table 2). No DCLK sequences were found in the genomes of invertebrate species (*D. melanogaster* and *C. elegans*). However, analysis of DCX proteins from *D. melanogaster* revealed a catalytic “protein C kinase-like” domain (IPR000719, PF00069) (Figure S2) located between residues 477–743. Based on these results, we performed a multiple-sequence alignment of all the retrieved DCX and DCLK sequences. Results revealed more than 50% sequence similarity between DCX and DCLK sequences (Table 2).

We then extracted the DCX1 conserved domain from DCX and DCLKs proteins, which was used to perform a multiple-sequence alignment. A similar analysis was conducted for DCX2 conserved domain. In both cases, a high conservation degree was observed between DCX and DCLKs proteins (Table 3). The same approach was applied to the kinase domain found in DCLK proteins from vertebrates, using the “protein C kinase-like” catalytic domain of the *D. melanogaster* DCX sequence as reference (Accession number: AAM11416) (Figure S2). Remarkably, results revealed that the DCX2 domain is much more conserved than DCX1 and the kinase domains among species analyzed, and the latter, appears to be not much conserved in the evolutionary scale (Table 3). Comparison of DCX1 and DCX2 domains of DCLK proteins from zebrafish and of DCX proteins from *A. burtoni* fish revealed a high similarity of sequence and structure (Table 3).

To verify the absence of DCX in the zebrafish genome, we performed a tblastn analysis using human DCX amino acidic sequence as query. Although no DCX orthologous genes were found on zebrafish, we found three loci putatively encoding different DCLK isoforms. Likewise, the genomes of the two invertebrate species did not show any conserved region corresponding to DCLK sequences. This supports the hypothesis that the DCLK-encoding gene is absent in invertebrates.

### 3.3. Western Blot Analysis Confirms the Presence of DCX in Burton’s Mouthbrooder Fish and Mice, but Not Zebrafish

**Figure 2:**
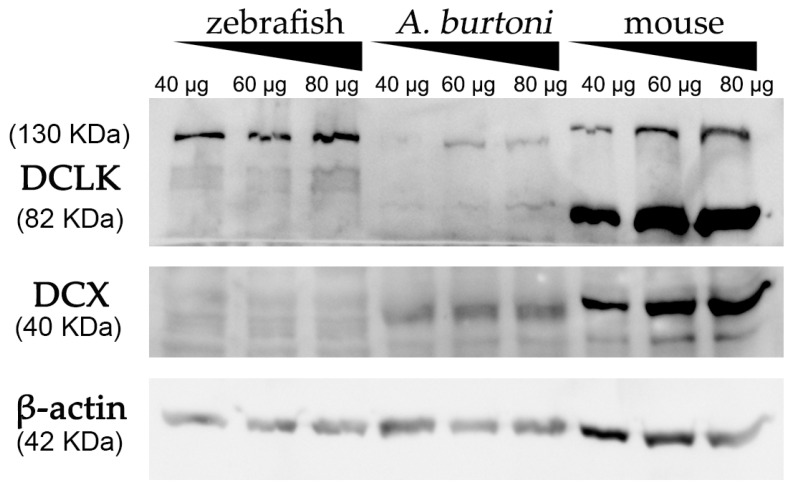
Western blot analysis of protein extracts derived from zebrafish, *A. burtoni*, and mice brains. Increasing concentrations of proteins were loaded in each well (40, 60 and 80 μg per well). Analysis shows the expression of DCX in *A. burtoni* and mice, but not in zebrafish extracts. β-actin was used as the loading control after the membrane stripping.

**Figure 3:**
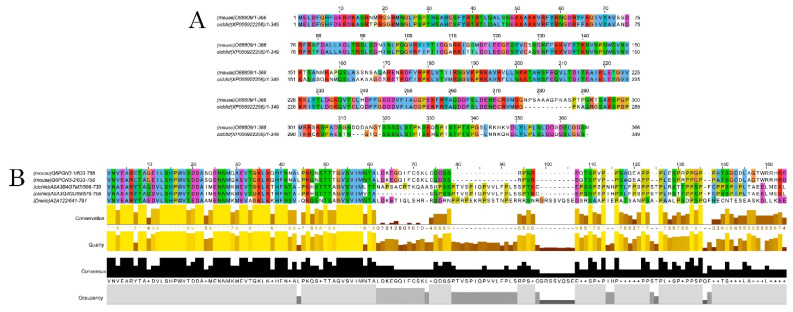
Alignments of DCX and DCLK amino acid sequences. Conserved residues between mice and *A. burtoni* DCX proteins (**A**); Conservation of the DCLK2 immunogen sequence use to detecting DCLK protein among the studied species (**B**).

Using a commercially available anti-DCX antibody, western blot assays revealed an intense and specific band of 40 KDa in protein samples from mice and *A. burtoni* fish (Figure 2 and Figure S3), which corresponded to the weight described for DCX protein (information related to sequence similarity between mice and *A. burtoni* DCX can be found in Figure 3A). No specific DCX band was found in zebrafish samples (Figure 2). A positive immunoreactive band for β-actin (loading control) in all samples proved that protein lysates from zebrafish brain were correct. In contrast, DCLK expression was found in protein lysates from the three studied organisms (Figure 2 and Figure S3); a strong immunoreactive band at 82 KDa is observed in lysates from mice brains, as well as a less intense band at 130 KDa. These two isoforms were also detected in samples from the *A. burtoni* brain, especially when 60 mg and 80 mg proteins were loaded, although the expression of the 82 KDa isoform is significantly lower. In the case of zebrafish, a specific immunoreactive 130 KDa band was found for the three tested protein concentrations (information related to conservation of the immunogen sequence among the studied species can be found in Figure 3B).

## 4. Discussion

Doublecortin (DCX) is a microtubule-binding protein expressed in differentiating and migrating neurons of the nervous system during embryonic and postnatal development [1,36]. Various reports have associated DCX to migrating cells, especially during the formation of cortical layers [1,3,37]. Our results indicate that DCX is also expressed in non-migrating neurons such as those located in the transition zone of the retina next to proliferating cells. In contrast, precursor cells giving rise to new rods are known to migrate but were negative for DCX. This is consistent with our previous finding in the fish telencephalon, where DCX is expressed by new neurons but not in migrating cells [38].

DCX gene expression have been described in different species, including invertebrates [29,39,40,41]. Apart of the MTs association, Friocourt et. al. identified and characterized a DCX-interacting protein in *D. melanogaster*, which is involved in cleaving ubiquitin from protein-ubiquitin conjugates [42]. In adults of the afrotherian tenrecs, DCX was shown to be expressed in the paleocortex [43,44]. In adult mammals, DCX is highly expressed in newly produced cells in the neurogenic zones; the subventricular zone along the lateral ventricle and the subgranular zone of the dentate gyrus [7,45]. Indeed, DCX has been adopted as a marker for neuronal precursors during adult neurogenesis [46]. In the visual system, DCX positive cells were found in the retinas of rats [47,48], sharks [5], teleost fish [6,23], and lampreys [4]. Here, we support the use of DCX as a marker for maturing neurons in the *A. burtoni* visual system. However, these results might not be extensive to all species since we were unable to find DCX in zebrafish in any of our experiments. This might suggest that DCX function could be compensated by other proteins.

An important question for understanding DCX function concerns its intracellular localization, since DCX may act similarly to classical MAPs and influence microtubule stability [49]. Although mutations in human DCX produces several defects in neuronal migration, a genetic deletion of DCX in mice causes a milder deformity [11,12,41]. Deuel et al. identified a different locus, doublecortin-like-kinase (DCLK), that encodes a protein with a similar “doublecortin domain” and microtubule stabilization properties that could compensate DCX function in rodents [50,51,52]. DCX and DCLK could directly or indirectly regulate microtubule-based vesicle transport, a process critical to both axon growth and neuronal migration [41,51,53,54].

The high level of conservation found in the evolutionary branches [11,29,41] and the very specific neuronal cell type that express DCX [2,45,46,55] would make this protein an excellent marker to detect neurogenesis events, not only during development but also in adulthood. It could be suggested that the absence of DCX in zebrafish could be compensated by DCLK. However, the exact compensatory mechanism and its implications during neuron migration and microtubule dynamics would require further investigation. The absence of DCX in zebrafish genome in addition to the lack of a corresponding amino acid sequence raises the question of how neurogenesis in zebrafish compensates for the missing DCX protein. This might ultimately help in investigating and better understanding neurogenesis in the vertebrate CNS.

## 5. Conclusions

The different techniques applied to detect the DCX protein in the visual system of teleost fish allowed us to determine the expression in *A. burtoni* but not in *D. rerio*. This work brings to light the high conservation of DCX sequences between different evolutionary groups, which makes DCX an ideal marker to study neurogenesis in various species. In *A. burtoni*, DCX is very useful for identifying new neurons generated in the retina and to follow their axons through components of the fish visual system. In addition, in teleosts, DCX apparently is not associated to migrating cells but could be related to axonal growth. Taking together, DCX represents an excellent marker for neurogenesis of most but not all animal models.

## Figures and Tables

**Table 1:** Database of 159 amino acidic sequences selected to perform protein alignments. Different species are used to compare DCX and DCLK sequence identity. From this database we also extract DCX1 and DCX2 conserved domains for further sequence analyses.

Protein and Species	Accession Number	Protein and Species	Accession Number	Protein and Species	Accession Number
DCLK from Kanglang fish	ROJ66296	DCLK from Human	NP_004725	DCLK from Zebrafish	AAI63926
DCLK from Kanglang fish	ROL01462	DCLK from Human	XP_016876336	DCLK from Zebrafish	AAI68500
DCLK from Kanglang fish	ROL42582	DCLK from Human	XP_016876337	DCLK from Zebrafish	BAF45322
DCLK from Kanglang fish	ROL54251	DCLK from Medaka	XP_004075345	DCLK from Zebrafish	BAF45323
DCLK from Betta fish	XP_029027475	DCLK from Medaka	XP_011474963	DCLK from Zebrafish	BAF45324
DCLK from Betta fish	XP_029027480	DCLK from Medaka	XP_011474968	DCLK from Zebrafish	BAF45325
DCLK from Betta fish	XP_029027952	DCLK from Medaka	XP_011474974	DCLK from Zebrafish	BAF45326
DCLK from Betta fish	XP_029027953	DCLK from Medaka	XP_011480649	DCLK from Zebrafish	NP_001128593
DCLK from Betta fish	XP_029027954	DCLK from Medaka	XP_011481611	DCLK from Zebrafish	NP_001139259
DCLK from Betta fish	XP_029027955	DCLK from Medaka	XP_011485544	DCLK from Zebrafish	NP_001139260
DCLK from Betta fish	XP_029030116	DCLK from Medaka	XP_020563782	DCLK from Zebrafish	NP_001139261
DCLK from *A. burtoni* fish	XP_005926513	DCLK from Medaka	XP_020564443	DCLK from Zebrafish	XP_005172728
DCLK from *A. burtoni* fish	XP_005926514	DCLK from Medaka	XP_024115177	DCLK from Zebrafish	XP_009290868
DCLK from *A. burtoni* fish	XP_005928154	DCLK from Medaka	XP_024143878	DCLK from Zebrafish	XP_009303718
DCLK from *A. burtoni* fish	XP_005935114	DCLK from Mouse	AAH21354	DCLK from Zebrafish	XP_009303720
DCLK from *A. burtoni* fish	XP_005935115	DCLK from Mouse	AAH50903	DCLK from Zebrafish	XP_021325742
DCLK from *A. burtoni* fish	XP_005935116	DCLK from Mouse	AAH64783	DCLK from Zebrafish	XP_021334856
DCLK from *A. burtoni* fish	XP_005951753	DCLK from Mouse	AAI33686	DCX from Betta fish	XP_029029871
DCLK from *A. burtoni* fish	XP_005951754	DCLK from Mouse	AF155819_1	DCX from Chick	AF330009
DCLK from *A. burtoni* fish	XP_014186335	DCLK from Mouse	NP_001104521	DCX from Chimp	PNI40040
DCLK from *A. burtoni* fish	XP_014190393	DCLK from Mouse	NP_001104522	DCX from *A. burtoni* fish	XP_005922256
DCLK from *A. burtoni* fish	XP_014190900	DCLK from Mouse	NP_001104523	DCX from Cow	NP_001193894
DCLK from *A. burtoni* fish	XP_014190901	DCLK from Mouse	NP_001182467	DCX from Dog	XP_022271525
DCLK from *A. burtoni* fish	XP_014190902	DCLK from Mouse	NP_001182468	DCX from Dog	XP_022271526
DCLK from *A. burtoni* fish	XP_014190903	DCLK from Mouse	NP_001344395	DCX from Dog	XP_022271527
DCLK from *A. burtoni* fish	XP_014190904	DCLK from Mouse	NP_001344397	DCX from Dog	XP_022271528
DCLK from *A. burtoni* fish	XP_014190905	DCLK from Mouse	NP_001344398	DCX from Dog	XP_022271529
DCLK from *A. burtoni* fish	XP_014190906	DCLK from Mouse	NP_001344404	DCX from Dog	XP_022271530
DCLK from *A. burtoni* fish	XP_014190907	DCLK from Mouse	NP_001344405	DCX from Dog	XP_022271531
DCLK from *A. burtoni* fish	XP_014193189	DCLK from Mouse	NP_064362	DCX from Dog	XP_022271532
DCLK from *A. burtoni* fish	XP_014193190	DCLK from Mouse	Q9JLM8	DCX from Fly	AAM11416
DCLK from *A. burtoni* fish	XP_014193191	DCLK from Mouse	XP_006501044	DCX from Glassy fish	XP_028278320
DCLK from Codfish	XP_030217849	DCLK from Mouse	XP_006501045	DCX from Glassy fish	XP_028278321
DCLK from Glassy fish	XP_028258882	DCLK from Mouse	XP_006501046	DCX from Glassy fish	XP_028278322
DCLK from Glassy fish	XP_028258890	DCLK from Mouse	XP_017174936	DCX from Human	AAC31696
DCLK from Glassy fish	XP_028258898	DCLK from Mouse	XP_030108271	DCX from Human	AAC31797
DCLK from Glassy fish	XP_028258907	DCLK from Mouse	XP_036018776	DCX from Human	AAC52037
DCLK from Glassy fish	XP_028275059	DCLK from Rat	NP_445795	DCX from Human	AAH27925
DCLK from Glassy fish	XP_028275060	DCLK from Tibetan fish	KAA0702164	DCX from Human	CAA05867
DCLK from Human	AAI52457	DCLK from Tibetan fish	KAA0704105	DCX from Human	CAA06617
DCLK from Human	NP_001317000	DCLK from Tibetan fish	KAA0710366	DCX from Human	NP_001182482
DCLK from Human	NP_001317001	DCLK from Tibetan fish	KAA0722200	DCX from Human	NP_001356299
DCX from Human	NP_001356300	DCX from Mouse	AAT58219	DCX from Rat	AAG18479
DCX from Human	NP_001356301	DCX from Mouse	AF155820_1	DCX from Rat	NP_445831
DCX from Human	NP_835364	DCX from Mouse	BAA33387	DCX from Rat	Q9ESI7
DCX from Human	NP_835365	DCX from Mouse	NP_001103692	DCX from Rat	XP_006257444
DCX from Human	NP_835366	DCX from Mouse	NP_001103693	DCX from Rat	XP_006257447
DCX from Human	O43602	DCX from Mouse	NP_001103694	DCX from Rat	XP_006257448
DCX from Killifish	AEY83972	DCX from Mouse	NP_034155	DCX from Rat	XP_017457656
DCX from Medaka	XP_023818290	DCX from Mouse	O88809	DCX from Shark	AFP00992
DCX from Mouse	AAC31799	DCX from Mouse	XP_006528761	DCX from Tibetan fish	KAA0710823
DCX from Mouse	AAH56391	DCX from Mouse	XP_030107072	Synapsin from Zebrafish	BAH84839
DCX from Mouse	AAH57010	DCX from Mouse	XP_030107073		
DCX from Mouse	AAH62974	DCX from Mouse	XP_030107074		

**Table 2:** Sequence similarities between selected proteins containing DCX1 and DCX2 domains. Comparison of entire DCX and/or DCLK amino acid sequences to assess protein conservation between different species.

Protein	Organism	Number of Aligned Sequences	Similarity
DCX	Human + Shark	15	96.7%
	Mammals	47	89%
	Mammals + *A. burtoni* fish	48	88.8%
	Mammals + Teleost Fish	58	83.3%
	Teleost Fish	11	82.5%
	Mammals + Teleost Fish + Drosophila + Shark + Chick	61	78.6%
DCLK	Mammals	30	55.9%
	Teleost Fish	71	53.5%
	Mammals + Teleost Fish	101	49.3%
DCLK from zebrafish + DCX from *D. melanogaster*		16	72.5%
DCLKs + DCX		159	50.1%

**Table 3:** Sequece similarities between DCX1 and DCX2 domains after its extraction from DCX and/or DCLK. Comparison of DCX1 and DCX2 conserved domains to analyze the identity between different species.

Domain	Organism	Number of Sequences	Similarity
DCX1	All	159	73.4%
DCX2	All	159	77.4%
Kinase	All	159	47.4%
DCX1	Teleost Fish	79	74.5%
DCX2	Teleost Fish	79	79.1%
Kinase	Teleost Fish	79	69.9%
DCX1	DCLK ZF + DCLK *A. burtoni* + DCX from another organisms	97	79.6%
DCX2	DCLK ZF + DCLK *A. burtoni* + DCX from another organisms	97	83.7%

## Data Availability

Not applicable.

## Supplementary Materials

The following are available online at https://www.mdpi.com/article/10.3390/biology11020248/s1, Figure S1: Scheme of DCX1 and DCX2 conserved domains found in DCX protein sequences. Figure S2: Scheme of the kinase conserved domain found in DCX protein sequences. Figure S3: Relative intensity quantification of western blot. File S1: Full Western Blot.
