# Supplementary material for: Doublecortin in the Fish Visual System, a Specific Protein of Maturing Neurons
Source: Biology (Basel). 2022 Feb 6;11(2):248. doi: 10.3390/biology11020248 (PMC8869232; doi:10.3390/biology11020248)
Supplement: Supplementary file 1 [file biology-11-00248-s001.zip › biology-1570120-Figures S1-S3.pdf]

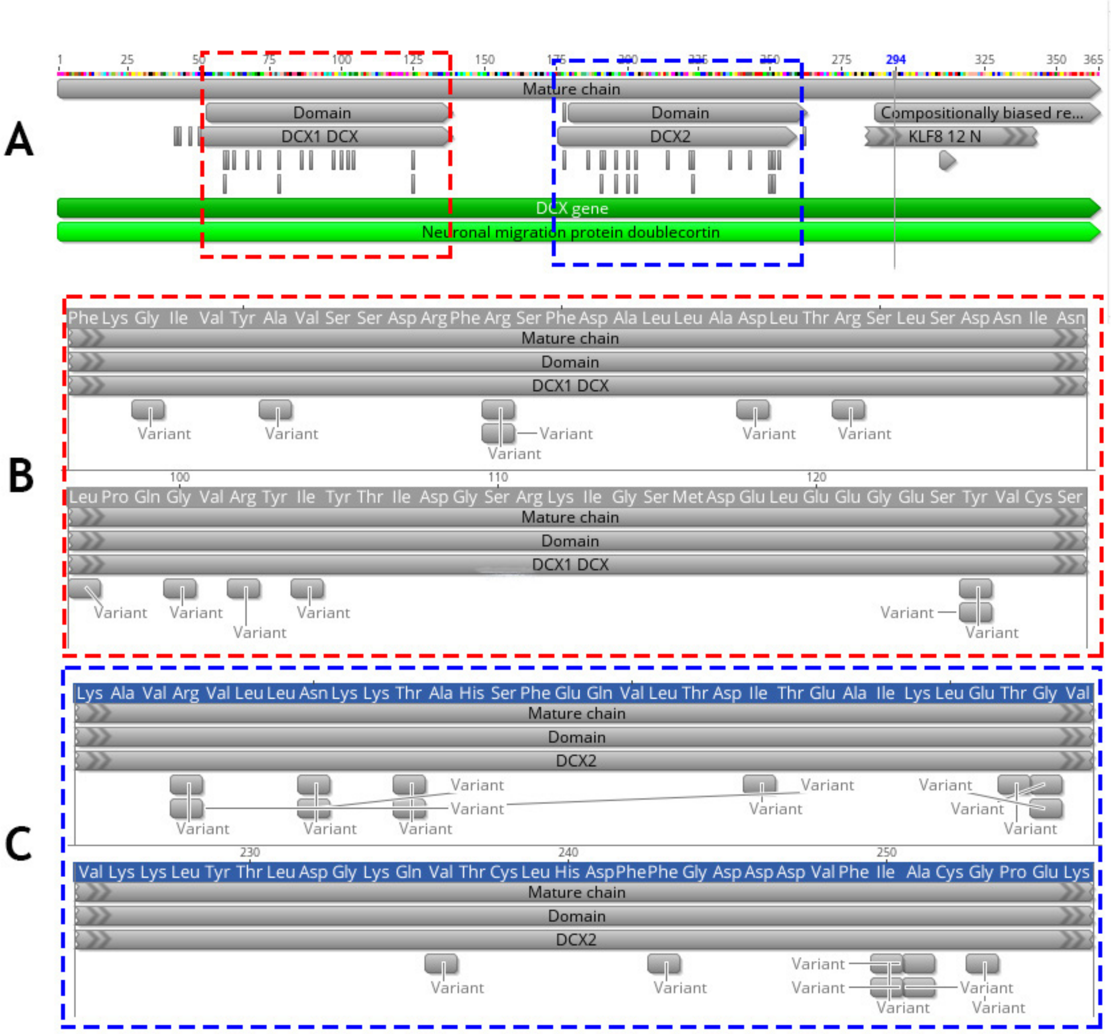

Figure S1. Scheme of DCX1 and DCX2 conserved domains found in DCX protein sequences.

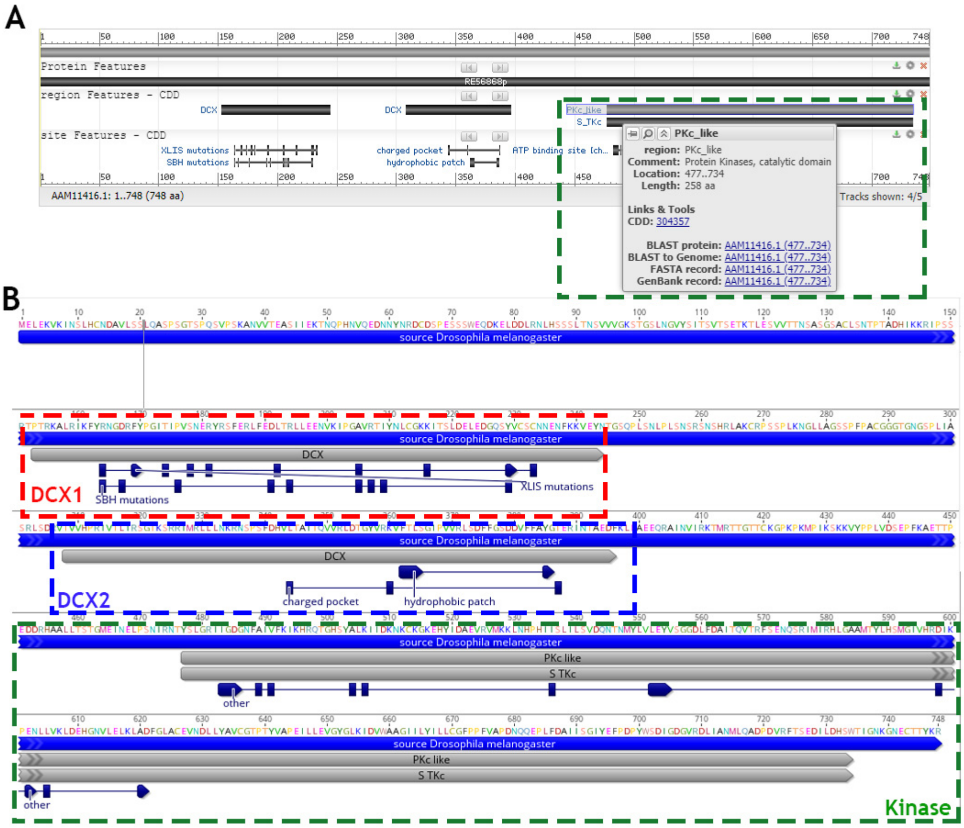

Figure S2. Scheme of the kinase conserved domain found in DCX protein sequences.

A

$$\text{Relative intensity (Test protein}/\beta\text{-actin)} = \frac{(Total\ D.O. - Background\ D.O.)_{test\ protein}}{(Total\ D.O. - Background\ D.O.)_{\beta-actin}}$$

	Protein (ug)	Total Optic Density					Specific Optic Density				Relative intensity		
		Actin	DCX	DCLK 130KDa	DCLK 82 Kda		Actin	DCX	DCLK 130KDa	DCLK 82 Kda	DCX/Actin	DCLK 130 / actin	DCLK 82 / actin
zebrafish	40	16185857	40643855	19156582		12407909	7174939	11362252	11397609	2771866	1.58	1.59	0.39
	60	15589623	36940370	17173109		15959291	6578705	7658767	9414136	6323248	1.16	1.43	0.96
	80	16496847	38551081	23129342		17825477	7485929	9269478	15370369	8189434	1.24	2.05	1.09
cichlid	40	20088671	49813144	13285433		16798924	11077753	20531541	5526460	7162881	1.85	0.50	0.65
	60	19857308	54829570	14745551		17555519	10846390	25547967	6986578	7919476	2.36	0.64	0.73
	80	19146423	55332691	12430076		19637246	10135505	26051088	4671103	10001203	2.57	0.46	0.99
mouse	40	23863742	54917665	12425891	11592839		14852824	25636062	4666918	6586767	1.73	0.31	0.44
	60	23576081	72910811	17643875	17248407		14565163	43629208	9884902	12242335	3.00	0.68	0.84
	80	18943011	83646641	20867863	22060219		9932093	54365038	13108890	17054147	5.47	1.32	1.72
Background		9010918	29281603	7758973	5006072	9636043							

2 different blanks because background intensity is different in the three samples, due to the fact that ECL signal is much brighter in the mouse samples

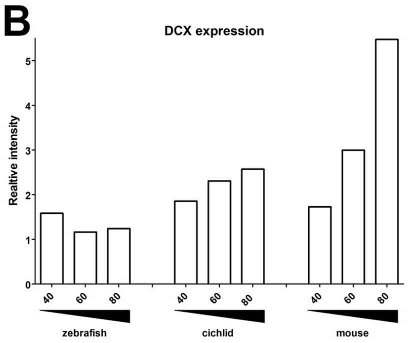

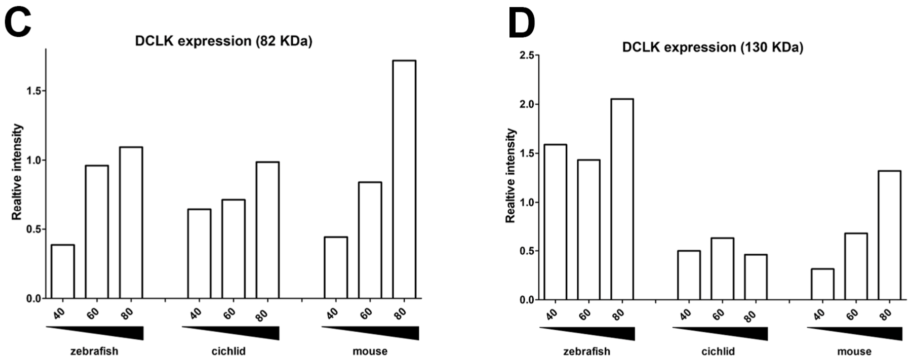

Figure S3. Relative intensity quantification of western blot.
