# Supplementary figures and images for: Doublecortin in the Fish Visual System, a Specific Protein of Maturing Neurons
Source: Biology (Basel). 2022 Feb 6;11(2):248. doi: 10.3390/biology11020248 (PMC8869232; doi:10.3390/biology11020248)

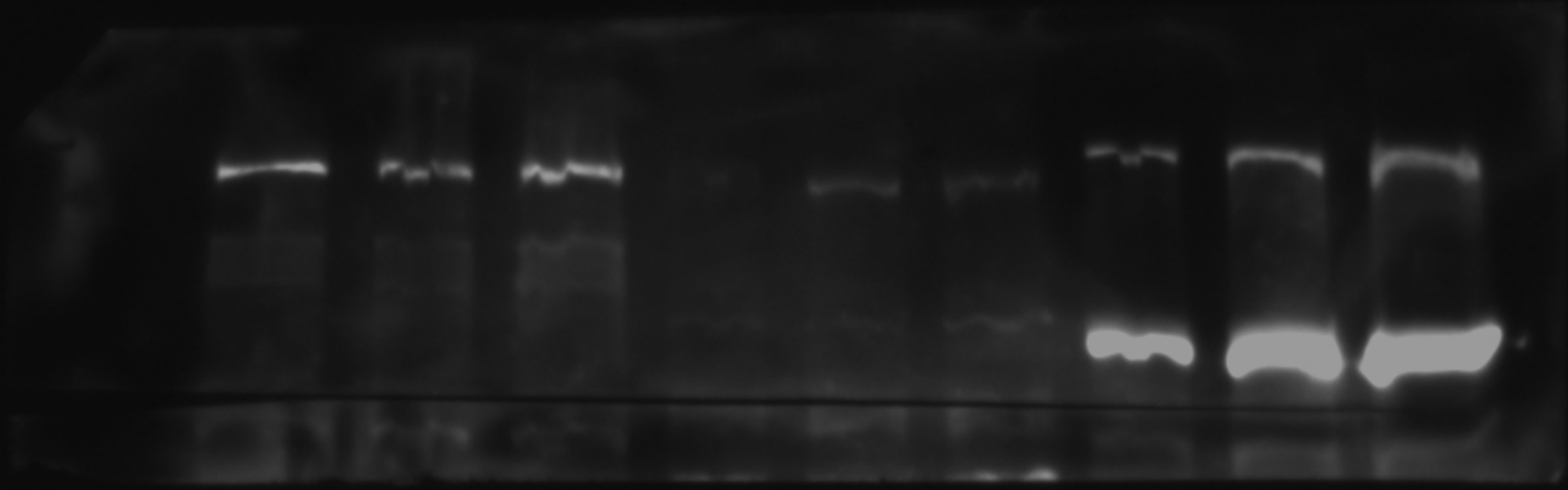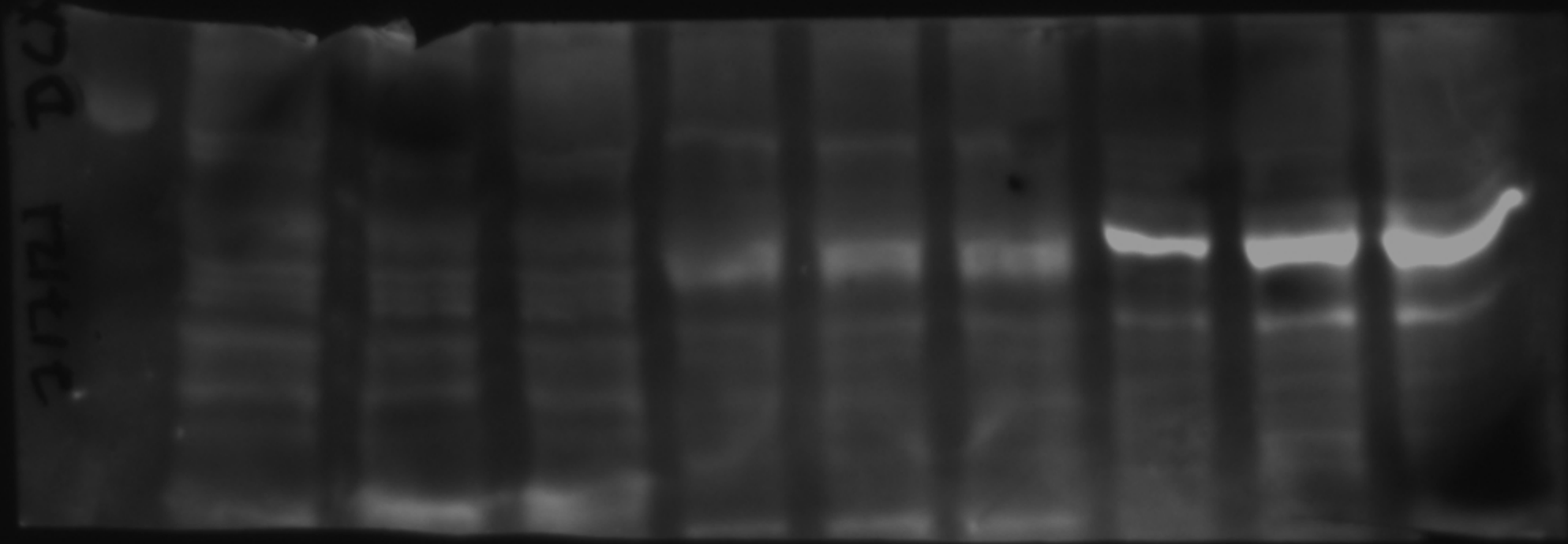

Supplement: Supplementary file 1 [file biology-11-00248-s001.zip › biology-1570120-File S1.pdf]
